# Data on prevalence of additive colors in local food and beverage products, Tehran, Iran

**DOI:** 10.1016/j.dib.2018.07.001

**Published:** 2018-07-09

**Authors:** Sahar Asadnejad, Ramin Nabizadeh, Abdullah Nazarinia, Gholam Reza Jahed, Mahmood Alimohammadi

**Affiliations:** a Department of Environmental Health Engineering, Food Safety Division, School of Public Health and Center for Environmental Research, Tehran University of Medical Sciences, Tehran, Iran; b Department of Environmental Health Engineering, School of Public Health and Center for Environmental Research, Tehran University of Medical Sciences, Tehran, Iran; c Manger of Laboratory of Kimia Test FAM, Tehran, Iran

**Keywords:** Artificial additive colors, Natural additives colors, Permitted colors, Non-permitted colors

## Abstract

The quality check and determination of permitted and non-permitted additive colors in food products is very important for customer׳s right protection and health. This survey was undertaken to demonstrate the frequently use of additive colors and products targeted to color adulteration in Iranian foods and beverages. From the 1120 of the samples, 18.86% contained artificial colors, 11.89% contained natural colors and 69.25% of samples had no additive colors. Tartrazine (E102) was the only non-permitted artificial dye used in samples. Among products with additive colors, only 4.38% of samples failed to meet with national Iranian standard and 61.23% of non-compliance samples were from non-industrial sectors and mostly were saffron and food containing saffron such as saffron rock candy and saffron chicken. These places and products quality are main the concern to solve the color adulteration in Iranian food market.

**Specifications Table**

**Table:** 

Subject area	Chemistry
More specific subject area	Food chemistry
Type of data	Tables
How data was acquired	The data were collected from Kimia Test Fam Laboratory, Tehran, Iran during the years of 2011–2015
Data format	Raw, Analyzed
Experimental factors	The mentioned parameters above, in abstract section, were analyzed according to the standard methods.
Experimental features	The food additive colors in various food products were determined.
Data source location	Tehran province, Iran
Data accessibility	The data is with this article and supplemented excel file

**Value of the data**

• The dataset shows the amount of additive colors in local food and beverage products.
• The dataset signifies the amount of permitted and non-permitted colors under Iranian laws.
• The dataset of this article identifies products with non-authorized colors, which can be useful for other investigators working in area of quality assurance of foods.

## 1 Data

The dataset provided here (Table 1, Table 2, Table 3) demonstrate the distribution of artificial and natural colors in samples, the distribution of artificial colors and non-compliance products with authorized and non-authorized colors according to Iranian national standard.

**Table 1:** Distribution of artificial and natural colors in products.

Category	Sample analyzed	Samples containing food colors	
		Artificial color	Natural color
Energy-Drink	83	71.08	28.92
Soft-drink	41	58.70	41.30
Candy/Gummy-candy	49	67.35	28.57
Chewing Gum	37	61.16	29.73
Saffron	72	13.89	1.39
Fruit drink powder	13	53.33	26.67
Rock-candy	46	13.04	17.39
Pastry/ready cake powder	34	17.65	11.76
Saffron Chicken	11	27.27	18.18
Edible ices	5	100	0
Snack	5	100	0
Fruit-drink	122	2.46	2.46
Syrup	17	16.67	55.56
Jelly	14	78.57	14.29
Chocolate/ice cream powder	5	40	20
Fruit-juice /fruit syrup	81	2.47	1.23
Sweet cream	8	0	87.50
Vinegar/vinegar drink	15	0	73.33
Ketchup sauce	19	5.26	0
Pomegranate sauce	6	0	33.33
Canned	41	0	22.44
Rusk powder	2	50	50
Cooked meat	3	33.33	0
Other products	391	0	0
Total	1120	18.86	11.89

**Table 2:** Distribution of artificial colors in products.

Category	E104%	E110%	E122%	E124%	E129%	E133%	E102%
Energy-Drink	0	0	0	0	1.2	0	1.2
Soft-drink	24.39	17.07	2.44	0	29.27	19.51	0
Candy/Gummy-Candy	8.16	8.16	0	0	0	2.04	0
Chewing Gum	0	0	0	0	0	11	0
Saffron	1.39	4.17	11.11	4.17	0	0	0
Fruit drink powder	23.08	0	0	0	46.15	23.08	0
Rock-candy	13.04	0	0	0	0	0	0
Pastry/ready cake Powder	0	0	0	0	7.14	0	0
Saffron Chicken	0	18.18	0	0	0	0	27.27
Edible ices	20	20	60	0	0	40	0
Snack	0	100	0	0	0	0	0
Fruit-drink	0	1.46	0	0	0	0	0.82
Syrup	0	0	0	0	11.11	5.56	0
Jelly	35.71	21.43	28.57	0	7.14	28.57	0
Chocolate/ice Cream Powder	20	20	20	0	0	20	0
Fruit-juice /fruit Syrup	1.23	0	1.23	0	0	0	0
Ketchup sauce	0	0	5.26	0	0	0	0
Cooked meat	0	33.33	0	0	0	0	0
Rusk powder	0	50	0	0	0	0	50
Total	24.83	20.69	13.79	2.07	15.86	18.62	4.14

**Table 3:** Distribution of permitted and non-permitted additive color in products.

Category	Polluted Products (%)	E104 (%)	E110 (%)	E122 (%)	E124 (%)	E129 (%)	E133 (%)	E102 (%)	E100 (%)	E150a-d (%)	Carthamus yellow (%)
Saffron Rock Candy	30.43	42.86	0	0	0	0	0	0	42.86	0	14.29
Saffron	15.28	6.67	20	53.33	20	0	0	0	0	0	0
Pastry	25	50	0	12.50	0	0	37.50	0	0	0	0
Saffron Chicken	45.45	0	40	0	0	0	0	60	0	0	0
Fruit Drink	2.48	0	66.67	0	0	0	0	33.33	0	0	0
Sauce	5.56	0	0	20	0	0	0	0	0	80	0
Cooked Meat	33.33	0	100	0	0	0	0	0	0	0	0
Energy drink	1.20	0	0	0	0	50	0	50	0	0	0
Fruit Juice	1.45	100	0	0	0	0	0	0	0	0	0
Fruit Syrup	16.67	0	0	50	0	50	0	0	0	0	0
Ready Cake Powder	10	0	0	0	0	100	0	0	0	0	0
Rusk Powder	50	0	50	0	0	0	0	50	0	0	0
Total	4.38	21.67	16.67	20	5	3.33	6.67	10	10	3.33	3.33

## 2 Experimental design, materials and methods

The Iranian national standard organization only permits seven artificial dyes of Quinoline Yellow (E104), Sunset Yellow FCF (E110), Azorubine (E122), as well as Ponceau 4R (E124), Allura Red AC (E129), Indigotine (E132) and Brilliant Blue FCF (E133) in different type of products. The prevalence use of permitted and non-permitted artificial dyes has been reported from different states of Iran, previously. The required data were collected from the results documented in Kimia Test FAM Laboratory during the five years of 2011–2015. The additive color of 1120 samples, which represent 30% of samples analyzed in Tehran city for additive colors, was determined using thin layer chromatography methods (TLC) according to 2634 Iranian national standard and were compared to 740 Iranian national standard for permitted food additive colors. The data were entered into an Excel spreadsheet and were analyzed by descriptive statistics [1], [2], [3], [4], [5], [6], [7], [8], [9], [10].
